# Facial Expression Recognition-You Only Look Once-Neighborhood Coordinate Attention Mamba: Facial Expression Detection and Classification Based on Neighbor and Coordinates Attention Mechanism

**DOI:** 10.3390/s24216912

**Published:** 2024-10-28

**Authors:** Cheng Peng, Mingqi Sun, Kun Zou, Bowen Zhang, Genan Dai, Ah Chung Tsoi

**Affiliations:** 1 School of Computing, Zhongshan Institute, University of Electronic Science and Technology of China, Zhongshan 528402, China; pengcheng@zsc.edu.cn; 2 School of Computer Science and Engineering, University of Electronic Science and Technology of China, Chengdu 611731, China; sun13624625009@163.com; 3 College of Big Data and Internet, Shenzhen Technology University, Shenzhen 518118, China; zhang_bo_wen@foxmail.com (B.Z.); daigenan@sztu.edu.cn (G.D.); 4 School of Computing and Information Technology, University of Wollongong, Wollongong, NSW 2522, Australia; moactsoi@gmail.com

**Keywords:** facial expression recognition, visual state space model, attention, object detection

## Abstract

In studying the joint object detection and classification problem for facial expression recognition (FER) deploying the YOLOX framework, we introduce a novel feature extractor, called neighborhood coordinate attention Mamba (NCAMamba) to substitute for the original feature extractor in the Feature Pyramid Network (FPN). NCAMamba combines the background information reduction capabilities of Mamba, the local neighborhood relationship understanding of neighborhood attention, and the directional relationship understanding of coordinate attention. The resulting FER-YOLO-NCAMamba model, when applied to two unaligned FER benchmark datasets, RAF-DB and SFEW, obtains significantly improved mean average precision (mAP) scores when compared with those obtained by other state-of-the-art methods. Moreover, in ablation studies, it is found that the NCA module is relatively more important than the Visual State Space (VSS), a version of using Mamba for image processing, and in visualization studies using the grad-CAM method, it reveals that regions around the nose tip are critical to recognizing the expression; if it is too large, it may lead to erroneous prediction, while a small focused region would lead to correct recognition; this may explain why FER of unaligned faces is such a challenging problem.

## 1. Introduction

Facial Expression Recognition (FER) plays a significant role in the areas of computer vision and affective computing. It deals with the automated detection and understanding of human facial expressions using algorithms. Due to the fast progression of deep learning technology, FER methods have seen substantial advancements, enabling more in-depth and precise emotional analysis. These improvements are visible not only in academic research but also in real-world applications, indicating immense potential. For example, FER technology is extensively utilized in social robots that can modify their behavior and dialogue by recognizing users’ facial expressions, providing more natural and personalized interaction experiences [1]. In addition, in mental health monitoring, FER technology helps identify and analyze patients’ emotional conditions, supplying vital supplementary information to medical experts. Some mental health applications leverage FER technology to track users’ mood changes and offer appropriate psychological counseling and support [2]. In driver safety systems, FER technology is used to identify driver fatigue and attention levels, issuing alerts when the driver is tired or distracted to prevent traffic accidents [3]. In the customer service industry, FER technology assists customer service systems by recognizing the emotional states of the customers, thereby providing more focused and empathetic services [4].

In conventional Facial Expression Recognition approaches, manually crafted features are essential. Geometric features entail detecting and pinpointing essential facial landmarks, such as the eyes, eyebrows, nose, and mouth, and evaluating the geometric relationships among these landmarks, such as distances, angles, and shapes. For instance, the distance between the mouth corners, the extent of eye openness, or variations in eyebrow angles can signify diverse emotional states. These geometric features constitute the foundation for the quantitative assessment of expressions, allowing the system to detect subtle facial variations. Appearance features concentrate on capturing the texture and intensity patterns of the face by scrutinizing slight changes in the skin surface and the distribution of light and shadow to identify expressions. Local Binary Pattern (LBP) is one of the frequently used techniques; it segments the image into small blocks and compares each pixel’s intensity values with its neighbors to seize texture characteristics [5]. The Histogram of Oriented Gradients (HOG) characterizes local shapes by computing the orientation and strength of image gradients [6], while Gabor filters capture facial detail features through filters of various frequencies and orientations [7].

Although they made early contributions, traditional visual-based FER techniques encounter several challenges. One major issue is their failure to fully capture the complexities and variations in human facial expressions. Handcrafted features often fail to represent subtle expression changes and variations due to lighting conditions, occlusions, and differences in individual facial structures. For example, changes in facial shadows and highlights under varying lighting conditions can significantly impact the efficiency of appearance-based feature extraction [7]. Furthermore, occlusion issues, such as wearing glasses, hats, or hand movements covering parts of the face, can introduce difficulties and errors in feature extraction [8]. These methods are typically noise-sensitive and require extensive preprocessing to achieve acceptable performance levels. To reduce noise impact, traditional methods often require preprocessing steps like image smoothing and histogram equalization. However, these steps not only increase computational complexity but can also result in the loss of certain expressive features [9]. As a result, in practical settings, traditional FER methods may perform poorly under uncontrolled and dynamic conditions. For instance, in outdoor environments, varying lighting conditions and complex backgrounds make it difficult for traditional FER techniques to operate reliably. Traditional FER methods usually depend on static images or video frames, ignoring the dynamic information of facial expressions. Facial expressions are typically continuous processes, and recognizing them based solely on single frames can lead to misinterpretations. For example, the initial and final stages of a smile may show similar facial features in a single frame, but they represent different emotional states. This limitation of static methods makes it challenging for traditional FER techniques to achieve high-precision expression recognition in dynamic environments [10].

The advent of deep learning technology has led to groundbreaking progress in FER. By building multi-layer neural networks, particularly Convolutional Neural Networks (CNNs), deep learning can automatically derive complex feature representations from raw data without the need for hand-crafted features [11]. This ability makes deep learning highly proficient in processing image data and detecting facial expressions. These techniques can autonomously learn hierarchical features from data, acquiring both low-level details and high-level semantic information. For example, CNNs identify features such as edges, textures, and shapes through several layers of convolution and grouping, and at higher stages, they grasp the semantic content of images [11]. This layered configuration allows deep learning models to thoroughly and deeply interpret image content, leading to more precise facial expression recognition. Another major advantage of deep learning methods is their efficient end-to-end training capacity. Traditional approaches generally involve distinct steps for face detection, feature extraction, and classification, whereas deep learning methods can blend these steps into a single cohesive model via end-to-end training. This amalgamation simplifies the processing workflow and enhances overall performance. The end-to-end training method not only minimizes the necessity for human intervention but also increases the uniformity and accuracy of the system, significantly boosting facial expression recognition results.

In this paper, we consider the challenging FER problem as a dual task end-to-end learning problem: the simultaneous detection of the face location and recognition of facial expressions registered on the captured face. As it is end-to-end, this would preclude dividing this problem into three separate stages: detection of the face in the image, aligning the face so that it is approximately in a frontal pose, and then performing classification. In an end-to-end operation, the detection and classification will be conducted simultaneously, without the separate step of aligning the detected face in an approximate frontal pose.

A popular simultaneous object detection and its classification framework is the YOLO (You Only Look Once) framework [12]. Broadly speaking, this framework consists of three parts: a preprocessing part, called a backbone network, a feature extraction part, called the neck network, and a classifier, with the coordinates of a group of bounding boxes, and a confidence estimate of the likelihood that the object is detected within a particular bounding box, called the head network [12]. In this paper, we wish to simultaneously detect and classify, but the input image must be of sufficiently high resolution to show minute details of the face to be detected.The backbone network would output multiscale signals $S_{i}$, $i=1,2,\ldots ,N_{S}$. The feature extraction part, the neck network, would extract features from each scale, and then output them to $N_{S}$ heads for joint classification and detection. As there are $N_{S}$ scale signals at the input of the feature extraction part, it is prudent to combine the signal at each scale with signals of all other scales, with those other scales suitably downsampled or upsampled so that they are compatible with the particular scale being considered at hand. Then, each of such combined signals at their individual scale would pass through a feature extractor: $F_{i}$, $i=1,2,\ldots ,N_{S}$. This multiscale feature extraction is called a Feature Pyramid Network (FPN) [12].

Conceptually, there are a number of candidates for the feature extractor $F_{i}$. For example, $F_{i}$ may consist of convolutional neural networks [12]; attention networks, e.g., channel attention, efficient channel attention, [13]; Mamba [14,15] may be considered as an input-dependent linear state space version of the self-attention mechanism [16]. Mamba may be considered as a good way to reduce the influence of the background around a pixel.

In this paper, we introduce a novel feature extractor $F_{i}$, $i=1,2,\ldots ,N_{S}$, which we call Neighborhood Coordinate Attention Mamba (NCAMamba). This is based on neighborhood attention (NA) [17], which extracts features that are related to the local neighborhood of the feature vector. In coordinate attention (CA) [18], each of the output channel pixels is the input channel pixel modulated independently by its horizontal direction relationships, and its vertical direction relationships. In NCA, it may be considered that each of the output channel pixels is the input channel pixel modulated independently by its horizontal direction relationships only in a neighborhood of the pixel, and the vertical direction relationships only in a neighborhood of the pixel. In other words, NCA only considers the local neighborhoods independently in the horizontal and vertical directions, while CA considers the entire horizontal axis and the entire vertical axis, respectively. The intuition we have is that for the human face, it is more beneficial to assume that the features at a particular location are dependent on its relationships with those features that are located within a small neighborhood in both horizontal and vertical directions. Mamba is a linear computational complexity input-dependent linear state space method originally designed for sequence-to-sequence modeling [14]. This has been extended to image processing, called VMamba [19], which involves four 2D selective scans (SS2D), resulting in a Visual State Space (VSS). Intuitively, VSS reduces the background information around a feature vector. Thus, combining NCA and VMamba in NCAMamba, introduced in this paper, combines the strengths of NCA in being able to enhance the feature vectors based on its local directional relationships in both the horizontal and vertical directions, and that of VMamba, in being able to reduce the background information around a feature vector.

Our feature extractor: NCAMamba consists of a parallel connection between the VMamba module, called VSS, and a squeeze-excite (SE) network, combined and then passed onto an NCA, before it is connected in a residual block with the input, which then passes onto another spatial attention, using NA instead of self-attention, an NA, followed by a channel attention module.

We applied this novel feature extractor in the FPN in the YOLOX framework to two unaligned face FER benchmark datasets, RAF-DB and SFEW, and found that on both datasets, our FER-YOLO-NCAMamba achieves significant improvements in the mean average precision (mAP) scores when compared with its nearest rival, FER-YOLO-Mamba [15].

Our main contributions are as follows:

- **Propose the Neighborhood Coordinate Attention:** To better capture local and global features in unaligned images, we introduced a new attention mechanism called Neighborhood Coordinate Attention (NCA). This mechanism effectively focuses on important local details within images while maintaining an understanding of the overall structure.
- **Integration of the Attention Mechanism with the Mamba Structure:** We innovatively combined NCA with the Mamba structure to propose the NCAMamba module. This module significantly enhances the model’s ability to capture global contextual information and long-range spatial dependencies, resulting in superior performance when processing complex images.
- **Development of the FER-YOLO-NCAMamba Architecture:** We integrated the NCAMamba module into the YOLOX architecture, forming a novel FER-YOLO-NCAMamba architecture. Through a series of experimental validations, we demonstrated the effectiveness and superiority of this architecture in practical applications, highlighting its potential in image feature extraction and object detection.

## 2. Related Work

In this section, we will briefly discuss the issues related to facial expression recognition (FER) in Section 2.1. The YOLO framework will be briefly discussed in Section 2.2, while the attention mechanism will be discussed in Section 2.3. Finally, a brief discussion of the ‘face in the wild’ FER datasets and the joint detection and classification problem to be studied is contained in Section 2.4.

### 2.1. Facial Expression Recognition

The Facial Expression Recognition (FER) domain has experienced notable progress lately, influenced by both traditional approaches and deep learning methodologies. Conventional FER techniques mainly depend on handcrafted features and established machine learning algorithms. For instance, Zhao et al. [20] suggested identifying facial landmarks and texture details using geometric and appearance attributes. Nonetheless, these techniques underperform when encountering variations in illumination, occlusion, and head movements. Recently, deep learning has markedly enhanced the effectiveness and robustness of FER. Khanzada et al. [21] and Zhou et al. [22] implemented Convolutional Neural Networks (CNNs) and multi-scale spatiotemporal networks for FER, adeptly managing diverse facial expressions and environmental changes via extensive datasets and sophisticated network designs. Furthermore, Yu et al. [23] investigated methods to boost FER precision through semi-supervised pretraining and temporal modeling, capitalizing on unlabeled data and temporal dependencies to increase the model’s generalization capacity.

### 2.2. YOLO

The YOLO (You Only Look Once) [12,24] architecture is highly regarded in end-to-end object detection and classification methods because of its effectiveness and speed. The YOLO architecture has three distinct parts: the preprocessing part, called the backbone network, which extracts features from the input image; a feature-enhancement part, called the neck network, in which the features extracted in the backbone network are further enhanced before being processed by a classification and detection part, called the head network [12]. The YOLO architecture has been adapted for Facial Expression Recognition tasks. Zhong et al. [13] illustrated the use of YOLOv5 in real-time FER, underlining its appropriateness for dynamic settings like driver monitoring systems and emotion-aware advertising. YOLOv5 [13] incorporates CSPDarknet53 (Cross Stage Partial Darknet53) as its backbone and utilizes the Path Aggregation Network (PANet) for improved feature extraction and fusion.

Other research has verified the efficiency of YOLO in FER applications. For instance, FER-YOLO merges YOLOv3 [25] with Squeeze-and-Excitation (SE) modules [26], assigning importance to each channel to better capture prominent facial features. Experimental outcomes indicate that FER-YOLO achieves a mean average precision (mAP) that is 3.03% superior to YOLOv3 on the RAF-DB dataset [27]. Another investigation suggested using YOLOv5 for face detection in conjunction with a shallow CNN model for expression recognition, reaching a 95.57% accuracy rate across seven distinct facial expressions [28].

### 2.3. Attention Mechanisms

In recent years, the integration of attention mechanisms [16] within computer vision has considerably advanced and become pivotal in numerous visual tasks. Originally, attention mechanisms were applied to computer vision to emulate the human visual system’s capacity to identify salient regions. This approach, regarded as a dynamic weight adjustment based on input image features, has been highly successful in various visual tasks, such as image classification, object detection, semantic segmentation, video comprehension, image generation, 3D vision, multimodal tasks, and self-supervised learning [29]. A notable development is the Neighborhood Attention Transformer (NAT) [17], which introduces the first efficient and scalable sliding window attention mechanism. NAT restricts self-attention (SA) to neighboring pixels, achieving linear time and space complexity without additional pixel movement, thus preserving translational invariance. Experimental findings indicate that NAT-based models excel in image classification and follow-up visual tasks. Further, in mobile network design, channel attention mechanisms like Squeeze-and-Excitation (SE) attention have shown effectiveness in enhancing model performance but often neglect positional information. To resolve this, Coordinate Attention (CA) [18] was introduced, which incorporates positional information into channel attention. This method divides channel attention into two 1D feature encoding processes, aggregating features along both spatial directions, achieving long-range dependency capture and maintaining precise positional information. Studies reveal that Coordinate Attention not only improves ImageNet classification but also outperforms in downstream tasks like object detection and semantic segmentation.

### 2.4. Face in the Wild Datasets

There are a number of ‘face in the wild’ benchmark datasets, e.g., AFEW, SFEW, MELD, AffWild, and RAF-DB [30]. These datasets are of various sizes and characteristics and intend to capture various aspects of faces occurring in real-world situations. There have been a variety of methods proposed in order to study these datasets. A good source of information concerning these datasets and some of the associated methods used to study them is contained in [30].

In this paper, we consider two ‘face in the wild’ datasets, RAF-DB, and SFEW, using advanced deep learning methods, which have been proposed only recently, e.g., Mamba [14], a linear computational complexity input-dependent state space; advanced attention mechanism, e.g., neighborhood attention (NA) [17]; coordinate attention (CA) [18]; and squeeze-excitation (SE) network [26], to solve the joint detection and classification problem. We chose Mamba because of its ability to reduce the influence of background information on the current feature vector. In ‘face in the wild’ datasets, this ability might be able to mitigate the effects of poor lighting conditions, i.e., occlusion. We chose to use NA because of its ability to process information within a local neighborhood. We chose to use CA because of its ability to process direction-aware information. We hope that by suitably combining the strengths of these four components, SE, NA, CA, and Mamba, and placing it in the FPN in the YOLO framework, a popular framework for simultaneous object detection and classification, we may be able to obtain reasonably good results on these two unaligned face in the wild datasets.

## 3. Method

In this paper, we study the joint task learning problem: the simultaneous detection and classification of facial expressions. We use the Yolo framework: a preprocessing part, the backbone network, the feature-enhancement part, the neck network, the classification and detection part, and the head network [12]. While our design effort is focused on the feature-enhancement part, we will mention the preprocessing part, the backbone network, and the head network briefly in Section 3.4. Our feature extractor, which we call NCAMamba (see Section 3.3) in the neck network, consists of four components: the VSS (Visual State Space) module (see Section 3.1), the squeeze-excitation (SE) module, the neighborhood coordinate attention (NCA) module, and a Remnant module consisting of an NA-Channel attention module (see Section 3.2). NCAMamba is formed by connecting the VSS block and SE block in parallel, and then they are combined before passing onto the NCA module. This output is then combined with a direct input from the input and passed to the Remnant block. The Remnant block consists of two residual networks connected in tandem, the NA block followed by the channel attention block. The overall network, consisting of the backbone, the neck, and the head network is described in Section 3.4.

### 3.1. Visual State Space

The VMamba (visual mamba) model [19] represents a new architecture that maintains linear complexity, preserving both global receptive fields and dynamic weights. It effectively mitigates the extensive computational demands linked with the quadratic complexity found in transformers [16]. A significant feature of vmamba is its VSS (visual state space) block, depicted in Section 3.1, inspired by the recently introduced state space models. The primary benefit of this structure is the attainment of linear complexity, with no loss in the global receptive field.

The VSS Block employs a 2D-Selective-Scan (SS2D) module inspired by the S6 (Selective Scan Mechanism). What sets S6 apart is its input-sensitive contextual nature, where dynamic weights are generated from the input information, promoting flexibility and adaptability within the mechanism. Consequently, the VSS Block can dynamically adjust its attention weights in response to various input characteristics, thus enhancing its focus on essential feature areas.

To mitigate direction sensitivity problems, the VSS Block includes a Cross-Scanning Module (CSM). The CSM examines input image features in four distinct directions (top-left to bottom-right, bottom-right to top-left, bottom-left to top-right, and top-right to bottom-left), converts these features into sequences, and subsequently consolidates them. This method guarantees that each pixel can assimilate contextual information from various directions, thereby improving the model’s capability to seize global information.

**Figure 1:**
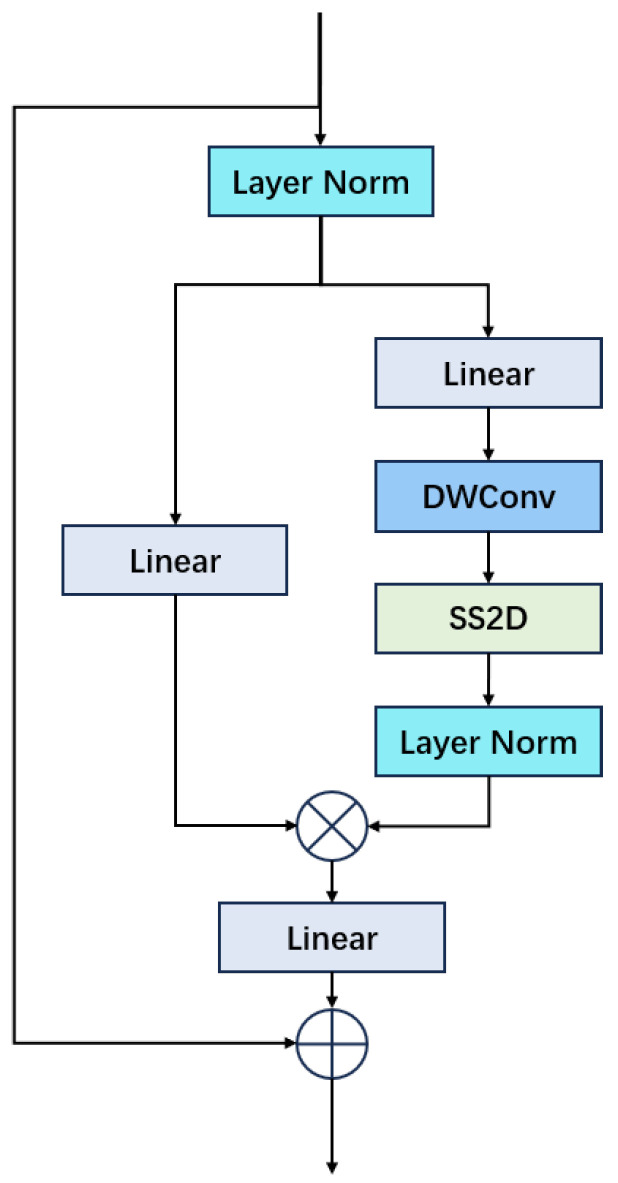
Illustration of VSS (Visual State Space) Block.

Mamba [14] was originally designed for 1D input-output sequence modeling using a continuous time-linear state space. This linear continuous time state space model can be discretized into a discrete time-linear state space representation through the use of a zero order hold. As it is a linear state space, it is possible to find a similarity transformation such that the **A** matrix in the state space model is diagonal, with entries >0 using what is known as Legende basis. Then, the associated **B** and **C** matrices in the state space representation would be learnable. As the **B** and **C** in the discrete time state space representation are dependent on a parameter, the discretization interval is δ. This discretization interval δ controls the extent to which the output is affected by the inputs. The output obtained by a particular input sequence is called a scan. So, by selectively scanning, it is possible to obtain different outputs. Conceptually, this can be thought of as another way of obtaining the output from the self-attention mechanism [16], the output of which (the value matrix) is in response to a query vector. In a way, one may consider the selective input as the query, and the linear state space model would then provide an output sequence according to this scan. The benefit of using linear state space is that it is linear in complexity, as compared to the quadratic complexity of the self-attention mechanism. This would allow a longer sequence to be considered, without incurring the quadratic complexity like in the case of self-attention. See Figure 1.

Now, for a 2D signal like an image, this may be converted into a sequence of patches using convolutional kernels with non-overlapping stride. Because it is a patch sequence derived from an image, each patch in the sequence would be corresponding to a particular location in the image. In this case, there exist four possible scan directions, from top left to bottom right using horizontal scan, from top left to bottom right using vertical scan, from bottom right to top left using horizontal scan, and from bottom right to top left using vertical scan. This is known as the 2D selective scan (SS2D) [19]. It is possible to have four different output sequences from these four scan possibilities. These four output sequences can be combined and reshaped back into an image. This is called a visual state space (VSS) [19]. Intuitively, for a particular block in the image, since in each of the four scans the block participates while its neighbors participate less, this will highlight the block while reducing the influence of its neighbors, which is similar to the type of processing performed by an edge detection filter.

### 3.2. Neighborhood Coordinate Attention

In our task of recognizing facial expressions, we aim to detect and categorize various emotions from unaligned facial images. The model must manage high-resolution images and simultaneously capture both local and global features. To improve the model’s capabilities, we incorporated the Neighborhood Attention (NA) mechanism into the C3 (Concentrated Comprehensive Convolution) module of YOLOX’s Feature Pyramid Network (FPN) [31]. NA directs each pixel’s attention to its immediate surroundings through an efficient and scalable sliding window mechanism. By confining each pixel’s self-attention to its closest neighbors, NA decreases the temporal and spatial complexity compared to traditional Self-Attention (SA), making it better suited for processing high-resolution facial images.

**Figure 2:**
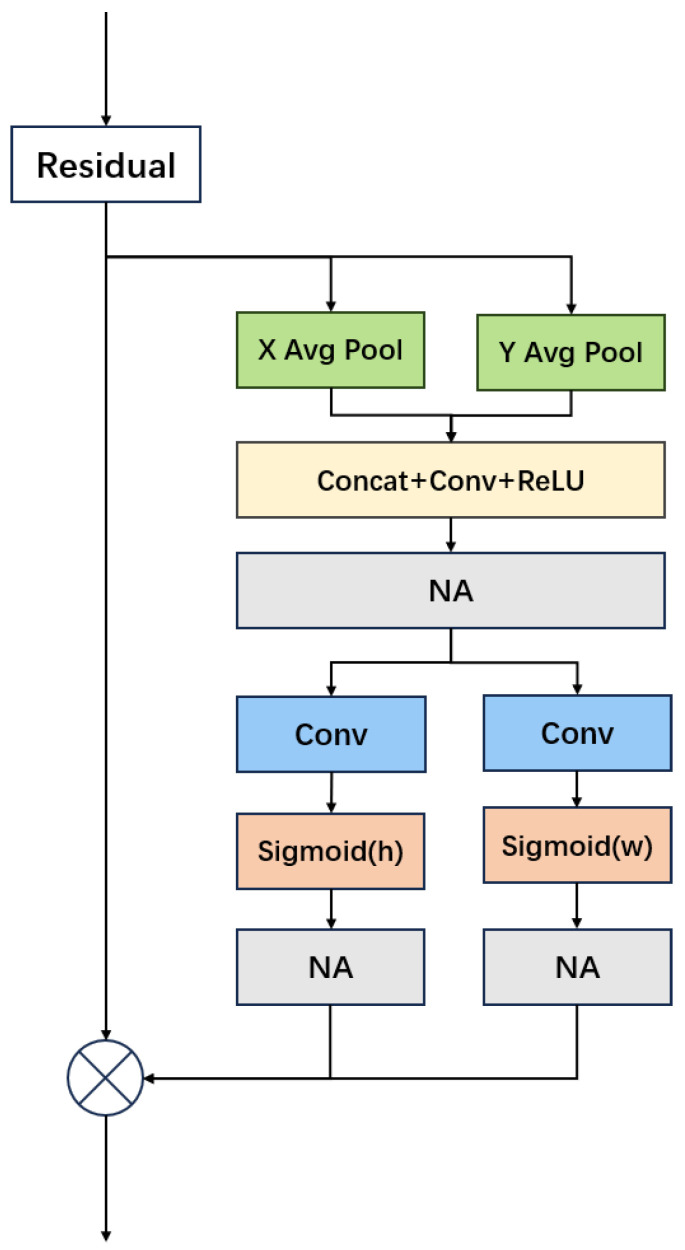
Illustration of Neighborhood Coordinate Attention module.

NCA comprises both Neighborhood Attention (NA) [17] and Coordinate Attention, illustrated in Figure 2. Note that here, we inserted three NA modules into the CA module, one in the middle and two, one each, in the horizontal direction, and one in the vertical direction, at the bottom of the CA module. CA captures channel relationships and long-range dependencies by utilizing precise positional information. It effectively gathers cross-channel as well as direction-aware and position-aware data. Typically, standard attention mechanisms, e.g., self-attention [16], tend to overlook positional details. CA addresses this by incorporating coordinate information, thus enhancing the model’s ability to handle positional cues. Unlike global attention mechanisms, NA emphasizes local neighborhood information, which better identifies local features in images or sequence data, and mitigates unnecessary long-range dependencies, enhancing the model’s robustness and stability. CA and NA mutually reinforce each other by targeting global and local information, respectively, improving the model’s capability to represent features. Both CA and NA are computationally efficient, and their combination further optimizes performance, ensuring the model delivers high results with increased computational efficiency.

Intuitively, what NCA is doing is that at each location on the face, it gathers information at the vicinity of this location, in both the horizontal direction and vertical direction. By comparison, what CA is doing, is at each location on the face, it gathers information along the entire horizontal direction and the entire vertical direction.

### 3.3. NCAMamba

**Figure 3:**
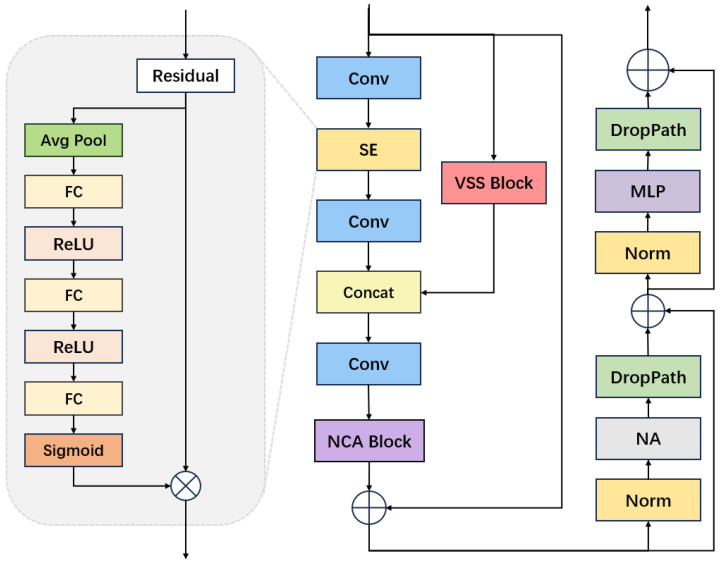
Illustration of NCAMamba architecture.

Within our task of facial expression recognition, we created a groundbreaking module specifically for this purpose, referred to as NCAMamba, depicted in Figure 3. NCAMamba combines the core VSS module from VMamba with an attention mechanism. Its overall design is influenced by the C3 architecture and is split into two branches. In one branch, we substituted the initial multiple bottleneck stack with the VSS structure. The VSS structure enables efficient feature extraction through the use of selective scanning mechanisms and adaptive weights, enhancing its capability to concentrate on key features.

### 3.4. Overall Architecture

In the other branch, a channel attention mechanism, a squeeze-excitation (SE) block, was implemented to automatically assess the significance of each feature channel. This method boosts relevant features while diminishing the less critical ones, thereby enhancing overall feature representation. Intuitively, the VSS block reduces the background influences on a patch, while the SE block highlights relevant features. Therefore, they are complementary to one another. The outputs from both branches are then concatenated and fed through a simple convolutional layer, followed by a residual connection, to ensure effective combination and retention of the learned features.

The merged output is subsequently processed by the NCA module, designed to extract global and local features more effectively. This NCA module integrates both the NA function and CA function, where the CA function captures detailed positional data and the NA function emphasizes local neighborhood data. As a result, the model’s capacity to manage global dependencies and local feature changes is enhanced. The output from the residual block, involving the VSS block, SE block, and NCA block, is then fed into two cascade attention blocks, the first one involving an NA in a residual block, and the second another residual block involving an MLP block and droppath strategy to reduce the risk of overfitting. For ease of reference, we will refer to these two residual blocks as the Remnant block. The Remnant block provides further enhancement of the features, as extracted by the NCA block.

**Figure 4:**
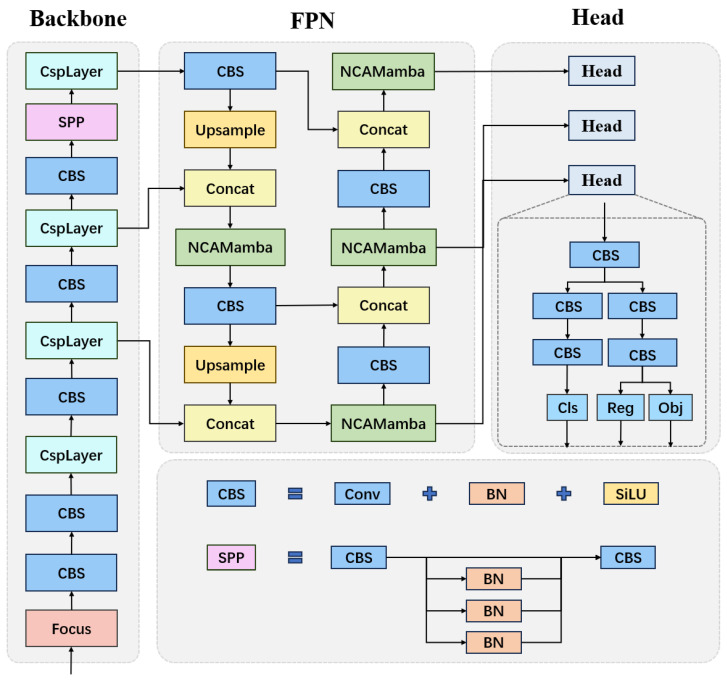
Illustration of Overall Architecture.

Overall, we adopted the YOLOX [31] framework (see Figure 4). The YOLOX architecture [31] comprises three essential parts: the Backbone, the Feature Pyramid Network (FPN), and the Head network. Together, these create a robust and efficient system for FER.

The backbone architecture of YOLOX is built upon CSPDarknet (Cross Stage PartialDarkNet). By utilizing the CSP Network technique [32], CSPDarknet [33] enhances both the efficiency of feature extraction and the network’s expressive ability. This method refines traditional deep convolutional networks by dividing and rearranging feature maps, thus lowering computational costs and boosting feature propagation. As a result, YOLOX is well-equipped to extract detailed facial expression features in large-scale datasets and complex environments, forming a robust backbone for ensuing detection operations.

As shown in Figure 4, the backbone network may be considered a multiscale signal generator: from an input image H×W×3, it outputs three signals: $S_{1}:10\times 10\times 512$, $S_{2}:20\times 20\times 256$, and $S_{3}:40\times 40\times 128$, respectively.

The Feature Pyramid Network (FPN) is essential in YOLOX, as it boosts the model’s capacity to identify facial expressions of varying sizes through multi-scale feature fusion. Since we have three scale signals, for this multiscale fusion to be possible, the scale signals would need to be suitably upsampled or downsampled so as to make them compatible. For instance, to fuse $S_{1}$ with $S_{2}$ and $S_{3}$, $S_{2}$ would need to downsampled to 10×10×512, and $S_{3}$ would also need to be downsampled to 10×10×512 and then fused with the $S_{1}$ signal to obtain an output of $S_{1}^{\prime }:10\times 10\times 512$. Similarly, it is possible to obtain fused output $S_{2}^{\prime }:20\times 20\times 256$, and $S_{3}^{\prime }:40\times 40\times 128$. These fused outputs then would be processed individually by an NCAMamba module, instead of the original C3 (Concentrated Comprehensive Convolution) structure in YOLOX [31].

YOLOX [31] uses a decoupled head arrangement, and it does not require any knowledge of anchors, unlike YOLOv3 or YOLOv5, which use a coupled head and require knowledge of the anchors. There are heads, each head with input $H_{i}\times W_{i}\times C_{i}$, where i=1,2,3. After passing through the first CBS block, with a 1×1 kernel, this is split into two branches: the classification branch and the regression branch. Both the classification branch and the regression branch go through 2 CBS blocks, each CBS block with 3×3 kernel. The classification branch then goes through another CBS block with 1×1 kernel, and outputs $H_{i}\times W_{i}\times C_{i}$ (cls). The regression branch, on the other hand, is further split into two branches, and each passes through a CBS block with 1×1 kernel. One branch outputs the regression (reg) with $H_{i}\times W_{i}\times 4$, while the other branch outputs $H_{i}\times W_{i}\times 1$, which gives the confidence of the bounding boxes (obj).

So, with an input image H×W×3, the output of the FER-YOLO-NCAMamba would be $H_{i}\times W_{i}\times C_{i}$ (cls), $H_{i}\times W_{i}\times 4$ (reg) and $H_{i}\times W_{i}\times 1$ (obj), i=1,2,3. These will go through a non-minimum suppression (NMS) (see, e.g., [34]) post-processing module, which will output a list of bounding boxes together with their category labels (label of expression), with the intersection over union (IoU) score below a preset threshold. The NMS will remove any duplicate bounding box (with an IoU score above the preset threshold).

## 4. Experiments

In this section, after a presentation of the characteristics of the datasets that will be used in the experiments (see Section 4.1), and information on some experimental details (see Section 4.2), we present a description of the metrics that will be used to assess the quality of the results obtained. Then, we present an ablation study on the relative importance of the major components in the NCAMamba module in Section 2. We then present a visualization analysis of the predictions in the application of FER-YOLO-NCAMamba model to two datasets, RAF-DB, and SFEW (see Section 4.5). Finally, we compare the performance of the FER-YOLO-NCAMamba model with other state-of-the-art methods in Section 4.6.

### 4.1. Datasets

In our research, we employed two datasets, RAF-DB (Real World Affective Faces Database) [35] and SFEW (Static Facial Expression in the Wild) [36], to train and assess our FER-YOLO-NCAMamba system. It is worth mentioning that we utilized the unaligned versions of these datasets, which play a crucial role in both the training and evaluation phases of the model. The distribution of the training and testing data subsets for both datasets is detailed in Table 1, illustrating the respective quantities for each set.

**RAF-DB**. The RAF-DB is an extensively utilized dataset that features a wide array of real-life facial expressions, including diverse emotional categories such as anger, disgust, fear, happiness, neutrality, sadness, and surprise. The dataset contains 15,339 facial images, each annotated with corresponding emotion labels. The RAF-DB sources its data from facial expression images found on the internet and captured in real-world settings, thus ensuring high authenticity and variety. In our research, we used the non-aligned version of RAF-DB, indicating that these images have not been subjected to standard alignment procedures, providing a more accurate depiction of facial expression variations in natural environments.
**SFEW**. The SFEW dataset concentrates on static snapshots of dynamic facial expressions, comprising 1251 images from films and video clips, displaying a variety of emotional states such as anger, disgust, fear, happiness, neutrality, sadness, and surprise. Like the RAF-DB dataset, the unaligned SFEW dataset has not been explicitly aligned, offering more authentic expression data from real-world scenarios. These unaligned SFEW images preserve the original facial features and expression variations in movies and videos, allowing the model to better handle the complexities of practical applications.

Leveraging unaligned dataset versions offers substantial benefits. Initially, unaligned images more accurately replicate diverse complex conditions encountered in real-world applications, including varied facial postures, lighting scenarios, and occlusions. This diversity in data strengthens the robustness and generalization capabilities of the model. Additionally, it removes the need for data alignment preprocessing, streamlining the data processing workflow and reducing computational expenses. Furthermore, unaligned data enable the model to better understand expression variations in natural contexts, enhancing its practical potential in real-world situations.

### 4.2. Implementation Details

The experiments detailed in this research were performed on a computer featuring an NVIDIA TeslaA40 GPU (Santa Clara, CA, USA), the PyTorch 2.0.0 deep learning framework and Python 3.11.9 For model training, the Adam optimizer was chosen with an initial learning rate of 0.001. Throughout the training phase, cosine annealing was utilized as the learning rate decay method to dynamically modify the learning rate, thereby improving the model’s convergence in the later training stages. The training process extended over 300 epochs, during which the model continuously refined the objective function to boost performance. We assigned a batch size of 15 for the SFEW dataset training and 100 for the RAF-DB dataset training.

The model’s backbone network was initialized using weights pre-trained on the COCO dataset [37], which endowed the model with strong feature extraction capabilities and sped up the training process. Using the extensive features learned from a large dataset, we improved the model’s performance in the FER task. All image inputs were standardized to be 320×320×3, according to the network’s input requirements. Furthermore, to improve the model’s robustness, we applied several simple data-augmentation techniques during training, such as random cropping, flipping, and rotation. We set the loU threshold in the NMS to 0.5.

### 4.3. Evaluation Metrics

In this study, our experiment is based on the YOLO architecture [31] for multi-class object detection and classification tasks, primarily using average precision (AP) as the evaluation metric. AP is used to measure the detection accuracy of the model at different thresholds, calculated by integrating the area under the precision–recall curve for each category. The specific calculation process is as follows:

1. **Precision and Recall Calculation**:

(1) $$\mathrm{Precision}=\frac{TP}{TP+FP}$$

(2) $$\mathrm{Recall}=\frac{TP}{TP+FN}$$

where TP represents the number of true positives, FP represents the number of false positives, and FN represents the number of false negatives.

It is possible to combine the precision and recall into an F1 score.

(3) $$F1=\frac{Precision\times Recall}{Precision+Recall}$$

2. **Average Precision (AP) Calculation**: average precision is the mean of precision values at different recall levels, calculated by integrating the precision-recall curve:

(4) $$AP=\int_{0}^{1}P\left(r\right)\;dr$$

where P(r) denotes the precision at recall *r*.

Likewise, it is possible to compute the average recall and average F1 accordingly.

3. **Mean Average Precision (mAP) Calculation**: The mean of the AP values for all categories is calculated as:

(5) $$mAP=\frac{1}{N}\sum_{i=1}^{N}AP_{i}$$

where *N* is the total number of categories and $AP_{i}$ is the average precision for the *i*-th category.

mAP comprehensively reflects the model’s detection performance across all categories and provides a thorough evaluation of the overall detection effect. During model evaluation, we assess its effectiveness and robustness in practical applications based on its AP performance on the validation and test sets.

### 4.4. Ablation Experiments

To assess the effectiveness of different components in our FER-YOLO-NCAMamba model, we conducted a series of ablation studies and tested them on the RAF-DB and SFEW datasets. Table 2 shows the results of this ablation study. The NCAMamba module consists of four components, with the SE block connected in parallel with the VSS block, and these, when combined, are connected with the NCA block. Then, the residual network formed by the input and the output of the NCA block passes through the Remnant block to obtain the output of the NCAMamba module. Because NCA lies on the main path between the input and output of the NCAMamba module, when this block is not required, its function is reduced to being an identity operation so as to maintain the connectivity between the input and output of this module.

The following observations could be made from Table 2:

- It is observed when both NCA and VSS are present in the NCAMamba module that the performances of both datasets are highest when compared with the situation when there is only a basic connection between the input and output: (SE block + I + Remnant block (row 1 for both datasets)).
- The RAF-DB dataset gives an impression of the relative importance between NCA and VSS blocks. Comparing rows 2 and 3 in Table 2, one notes that when VSS is present and NCA is absent, the mAP is 83%, while when NCA is present but VSS is absent, it achieves an mAP of 82.88%. This might signify that for this dataset, the presence of VSS would be more beneficial than the situation when NCA is present. Now, intuitively, the function of VSS is to reduce the influence of the background (surroundings around a particular location), and in this case, it seems to indicate that it is more beneficial to have VSS present than NCA present.
- The situation is different for the SFEW dataset. In this case, the presence of VSS is less beneficial than the presence of NCA. In other words, for this dataset, NCA is exerting more influence than VSS. Indeed, this dataset, with VSS present, achieves even lower performance than if this component is absent (comparing row 1 and row 3). In this case, one is tempted to interpret that the presence of VSS would be detrimental to the performance of the NCAMamba module.
- Comparing the situations with or without the presence of VSS on these two datasets, one gains an insight concerning the background information influence on the performance of the NCAMamba module: if the background is complex, then the VSS block or the like would need to be strengthened in order that it might function well in all situations. This might be an interesting future direction to study, especially in view of more powerful VSS and similar methods being introduced: multi-scale VMamba [38] and local VMamba [39].
- On the other hand, the NCA shows a more consistent trend. On both datasets, comparing rows 1 and 2, the situation with NCA present improves on when NCA is absent but replaced by an identity operation. Comparing row 2 and row 4 for each dataset, it is found that the presence of NCA and VSS further improve on the performance.

This observation allows us to conclude that NCA is relatively more important than VSS.

### 4.5. Visualization Analysis

**Figure 5:**
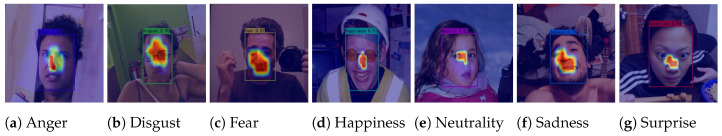
Detection results and corresponding heatmaps on the RAF-DB dataset, where the detection is correct.

**Figure 6:**
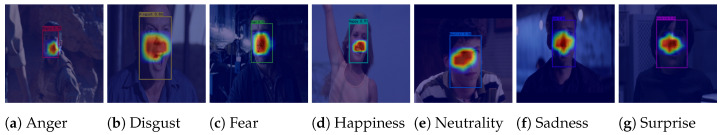
Detection results and corresponding heatmaps on the SFEW dataset, where the detection is correct.

**Figure 7:**
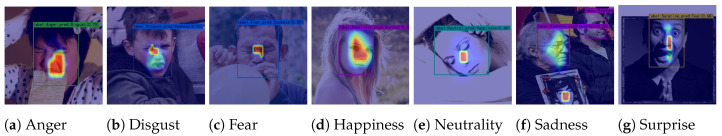
Detection results and corresponding heatmaps on the RAF-DB dataset, where the detection is incorrect.

**Figure 8:**
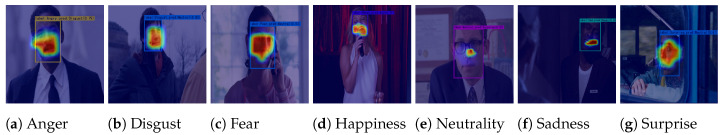
Detection results and corresponding heatmaps on the SFEW dataset, where the detection is incorrect.

In our study on visualization, we chose a representative image from each category in both the SFEW and RAF-DB datasets and conducted predictions on these images. Following the predictions, using the grad-CAM (gradient-based Class Activation Mapping) method [40], we superimposed the prediction boxes and heatmaps on the original images to visually represent the model’s detection and classification outcomes. These heatmaps pinpoint the key regions the model concentrates on. For example, in the “Neutral” category image, the heatmap reveals that the model focuses mainly on the central part of the face, which is essential for displaying a neutral expression. Figure 5 (RAF-DB) and Figure 6 (SFEW), respectively, depict instances with accurate predictions, whereas Figure 7 (RAF-DB) and Figure 8 (SFEW), respectively, show instances with incorrect predictions.

Some observations on Figure 5, Figure 6, Figure 7 and Figure 8 are as follows:

- It is clear that for the correctly predicted images in both RAF-DB and SFEW (see Figure 5 and Figure 6), the focus of the FER-YOLO-NCAMamba appears to be on the central region of the face, around the nose tip region. This confirms observations made elsewhere (see e.g., [41]).
- For the incorrectly predicted samples (see Figure 7 and Figure 8), it appears that the identified region is correctly around the nose tip, but in these cases, the identified region appears to be much larger than those correctly identified ones (see Figure 5 and Figure 6) for the single face image. If the image has more than one face, then it appears to be that the model focused on the wrong face (see Figure 7 ‘sad’).

From these observations, it may be concluded that the region around the nose tip would be crucial to FER. If the estimate of this region is too large, then this may lead to incorrect prediction. If the estimate of this region is small and focused, then this may lead to a correct prediction. However, because it is a visual device, it is therefore very difficult to quantify the meaning of ‘large’ and ‘small’. While no doubt this insight is very useful in explaining the failure or success in FER, it is difficult to leverage this insight to provide a more accurate prediction of facial expressions.

### 4.6. Comparison with State-of-the-Art Methods

Some observations on Table 3 and Table 4 are as follows:

- It is noted that on both datasets, the performances in terms of mAP % of our FER-YOLO-NCAMamba are superior when compared with those obtained by other state-of-the-art (SOTA) methods. In particular, it performs at 83.30 mAP when compared with its nearest rival: FER-YOLO-Mamba at 80.31 mAP on the RAF-DB dataset, while it achieves a 68.66 mAP when compared with 66.67 mAP achieved by FER-YOLO-Mamba on the SFEW dataset. Note that as the underlined datasets are unaligned, it would therefore be difficult to compare these results with those obtained by first performing some alignments on the samples before classification, as in such cases, the detection aspect would be largely irrelevant. But in such cases, the accuracy results would not be robust, as they are based on aligned information rather than on unaligned samples. The main difference between the FER-YOLO-Mamba architecture and the FER-YOLO-NCAMamba architecture lies in the fact that we include the NCA module and the Remnant block. Therefore, these results show the benefit of having some modules, which will consider the relationships between a feature vector at a location on the face, with those feature vectors in the neighborhood of the feature vector.
- It is observed in both Table 3 and Table 4 that the improvement of FER-YOLO-NCAMamba over that of FER-YOLO-Mamba or other SOTA methods is not uniform across all classes. For instance, on the SFEW dataset, FER-YOLO-NCAMamba performs relatively weaker than FER-YOLO-Mamba in the classes of Happiness, Sadness, and Surprise, while on the RAF-DB dataset, its performance is relatively lower than those of FER-YOLO-Mamba in the Disgust and Happiness classes, and in both cases, the differences are not large, <2%. This might be due to the very complex relationships between the background and the foreground (the face), which, as indicated in the Ablation study (see Section 4.4), the VSS component in our FER-YOLO-NCAMamba model is not very good at reducing the background information when compared with the foreground in the case of the SFEW dataset.
  Therefore, this opens the way to further improve the FER-YOLO-NCAMamba model, e.g., to make it multi-scale [38], so that it could take full advantage of the multi-scale nature of the underlying information, as expressions on the face could manifest themselves in small regions, and that these regions, while not contagious, are related. Alternatively, it might be possible to pay more attention to the local regions, using some ideas from LocalMamba [39]. As the results in this paper have shown the importance of being able to process the features around a small neighborhood, as this is what NA is good for, the CA indicates that directional information could also be important, so it just might be possible to extend LocalMamba [39] so that the Mamba could consider local information, as well as directional information. This would be a fruitful area to explore in future research.

**Table 3:** Performance comparison of different methods on the SFEW dataset, numbers highlighted with an underline represent our method’s highest performance in the same category.

Method	Year	*AP* (%)							*mAP* (%)
		Anger	Disgust	Fear	Happiness	Neutrality	Sadness	Surprise	
SSD [42]	2015	62.77	47.24	44.74	91.20	55.50	66.48	46.59	59.22
RetinaNet [43]	2017	68.91	58.59	55.23	81.87	43.10	64.16	24.86	56.67
YOLOv3 [25]	2018	19.52	0.00	5.88	50.28	37.45	21.11	0.00	19.18
CenterNet [44]	2019	39.54	0.00	25.12	68.57	22.14	43.95	0.00	28.48
EfficientNet [45]	2019	15.40	1.18	17.81	29.68	17.02	29.26	0.72	15.87
YOLOv4 [33]	2020	29.58	0.00	0.00	21.12	13.78	21.67	0.00	12.31
YOLOv5	2020	23.56	0.00	0.00	23.64	25.52	22.71	0.00	13.63
YOLOvX [31]	2021	67.01	73.86	70.48	90.81	36.15	70.26	39.55	64.02
YOLOv7 [46]	2022	57.47	64.64	52.55	74.34	32.44	48.44	32.26	52.02
YOLOv8	2023	56.24	45.24	53.76	87.50	33.48	44.69	42.68	51.94
FER-YOLO-Mamba [15]	2024	74.07	64.49	58.87	90.94	48.01	71.83	58.52	66.67
FER-NCAMamba (Ours)	2024	75.75	75.25	61.56	86.50	59.38	66.03	56.16	68.66

**Table 4:** Performance comparisons of different methods on the RAF-DB dataset, numbers highlighted with an underline represent our method’s highest performance in the same category.

Method	Year	*AP* (%)							*mAP* (%)
		Anger	Disgust	Fear	Happiness	Neutrality	Sadness	Surprise	
SSD [42]	2015	81.23	62.91	57.01	95.72	80.34	78.32	89.71	77.89
RetinaNet [43]	2017	82.07	53.74	53.56	94.63	80.13	77.50	87.80	75.63
YOLOv3 [25]	2018	58.01	28.56	37.02	88.09	67.89	63.78	72.59	59.42
CenterNet [44]	2019	53.26	17.41	30.62	91.32	75.36	66.83	84.63	59.92
EfficientNet [45]	2019	68.72	52.25	45.47	93.75	78.67	76.96	84.31	71.45
YOLOv4 [33]	2020	39.25	0.00	10.36	87.61	52.77	45.72	59.91	42.23
YOLOv5	2020	45.75	8.86	0.00	91.77	64.73	65.60	74.36	50.15
YOLOvX [31]	2021	78.38	62.40	57.85	96.82	80.45	83.35	89.56	78.40
YOLOv7 [46]	2022	62.20	55.80	44.72	92.01	73.20	74.72	74.57	68.17
YOLOv8	2023	74.50	50.40	50.85	93.33	76.39	76.30	82.89	72.09
FER-YOLO-Mamba [15]	2024	79.55	64.32	62.00	97.43	83.23	84.22	91.44	80.31
FER-NCAMamba (Ours)	2024	85.82	63.24	67.32	95.31	90.92	88.99	91.46	83.30

## 5. Conclusions

In this paper, we explore the solution to the joint detection and classification tasks in FER of unaligned images using a novel module, NCAMamba, as the main processing block in the Feature Pyramid Network (FPN) part of the YOLOX framework [31]. The NCA further enhances the combined features of the VSS block and the SE block by focusing on the local directional information in both the horizontal and vertical directions. Ablation studies on two benchmark-unaligned FER datasets, RAF-DB and SFEW, show the relative importance of VSS versus NCA; in this case, it shows that NCA is relatively more important than VSS. Visualization analysis using the grad-CAM method confirms that the NCAMamba module would concentrate more on the central regions around the nose tip and the upper lip for the correct predictions of facial expressions. Moreover, comparing the performance of the FER-YOLO-NCAMamba model with other state-of-the-art methods shows that FER-YOLO-NCAMamba achieves the best performance as measured by the mAP, significantly improving its nearest rival, FER-YOLO-Mamba. This achievement may be attributed to the capability of the NCA module in being able to enhance the features extracted by both the SE block and the VSS block, in being able to allow a feature vector to make use of the information in some local neighborhood in both the horizontal and vertical directions.

There are a number of directions in which the performance of the FER-YOLO-NCAMamba model could be improved. These include data augmentation, as we currently use very simple data-augmentation schemes. It may be possible to use masked autoencoder (MAE) [47] or its multi-scaled version [48], which randomly masks up to 75% of the input image, and then reconstructs the masked image. This will make the model more robust, and this could be useful, especially for small datasets like SFEW. A second direction of future research would be to use more sophisticated VMamba models, e.g., using a multi-scale version of VMamba [38], or one that will make use of the local information more effectively, like LocalVMamba [39]. A third direction of future research would be to consider making use of the information available in a batch-processing situation. The input to the joint detection and classification problem actually consists of B×H×W×3, where *B* is the batch size and H×W is the dimension of the image. In this paper, we only make use of H×W×3 and do not make use of the batch information explicitly. Some very recent work on FER of aligned faces shows that making use of this batch information, which they called batch transformer network (BTN) [49], achieves some of the best results when compared with other SOTA methods. It may be possible to adapt the BTN within the FER-YOLO-NCAMamba model and obtain improved results.

## Figures and Tables

**Table 1:** Summary of the datasets used in our experiments.

Dataset	Train	Test	Classes
RAF-DB	12,271	3068	7
SFEW	1125	126	7

**Table 2:** Ablation Experiments. SE denotes the branch which consists of Conv + SE + Conv; the NCA and NA modules have a neighborhood size of 7×7, the Remnant block consists of the two residual blocks after the NCA module, underlined numbers denote the peak values achieved and ✓ indicates that the module is included in the model.

Dataset	Methods				mAP(%)	Avg_F1	Avg_Recall (%)	Avg_Precision (%)
	SE	VSS	NCA	Remnant				
SFEW	✓		see Note 1	✓	66.81	0.63	61.95	67.71
	✓		✓	✓	67.39	0.61	62.27	65.20
	✓	✓	see Note 1	✓	64.35	0.61	64.10	61.54
	✓	✓	✓	✓	68.66	0.59	58.49	70.13
RAF-DB	✓		see Note 1	✓	82.37	0.77	75.47	78.46
	✓		✓	✓	82.88	0.78	76.86	78.84
	✓	✓	see Note 1	✓	83.00	0.79	76.31	81.00
	✓	✓	✓	✓	83.30	0.78	76.50	80.74

Note 1: The NCA module is reduced to an identity operation.

## Data Availability

Data are contained within the article.
